# Incremental prognostic value of left atrial and biventricular feature tracking in dilated cardiomyopathy: a long-term study

**DOI:** 10.1186/s12968-023-00967-4

**Published:** 2023-12-07

**Authors:** Xiaorui Xiang, Yanyan Song, Kankan Zhao, Shiqin Yu, Shujuan Yang, Jing Xu, Jiaxin Wang, Zhixiang Dong, Xuan Ma, Zhuxin Wei, Yun Tang, Minjie Lu, Shihua Zhao, Xiuyu Chen

**Affiliations:** 1 MR Center, Fuwai Hospital, National Center for Cardiovascular Diseases, Chinese Academy of Medical Sciences and Peking Union Medical College, Beijing, 100037 China; 2 Paul C. Lauterbur Research Center for Biomedical Imaging, Shenzhen Institutes of Advanced Technology, Chinese Academy of Sciences, Shenzhen, 518055 China

**Keywords:** Dilated cardiomyopathy, Cardiovascular magnetic resonance, Feature tracking, Myocardial strain

## Abstract

**Background:**

Despite the use of cardiovascular magnetic resonance (CMR) feature tracking (FT) imaging to detect myocardial deformation, the optimal strain index in dilated cardiomyopathy (DCM) is unclear. This study aimed to determine whether atrial and biventricular strains can provide the greatest or joint incremental prognostic value in patients with DCM over a long follow-up period.

**Methods:**

Four hundred-twelve DCM patients were included retrospectively. Comprehensive clinical evaluation and imaging investigations were obtained, including measurements of CMR-FT derived left atrial (LA) reservoir, conduit, booster strain (εs, εe, εa); left ventricular (LV) and right ventricular (RV) global longitudinal, radial, circumferential strain (GLS, GRS, GCS). All patients were followed up for major adverse cardiac events (MACE) including all-cause mortality, heart transplantation, and implantable cardioverter defibrillator discharge. The predictors of MACE were examined with univariable and multivariable Cox regression analysis. Subsequently, nested Cox regression models were built to evaluate the incremental prognostic value of strain parameters. The incremental predictive power of strain parameters was assessed by Omnibus tests, and the model performance and discrimination were evaluated by Harrell C-index and integrated discrimination improvement (IDI) analysis. Patient survival was illustrated by Kaplan–Meier curves and differences were evaluated by log-rank test.

**Results:**

During a median follow-up of 5.0 years, MACE were identified in 149 (36%) patients. LAεe, LVGLS, and RVGLS were the most predictive strain parameters for MACE (AUC: 0.854, 0.733, 0.733, respectively). Cox regression models showed that the predictive value of LAεe was independent from and incremental to LVGLS, RVGLS, and baseline variables (HR 0.74, 95% CI 0.68–0.81, *P* < 0.001). In reclassification analysis, the addition of LAεe provided the best discrimination of the model (*χ*^2^ 223.34, *P* < 0.001; C-index 0.833; IDI 0.090, *P* < 0.001) compared with LVGLS and RVGLS models. Moreover, LAεe with a cutoff of 5.3% further discriminated the survival probability in subgroups of patients with positive LGE or reduced LVEF (all log-rank *P* < 0.001).

**Conclusion:**

LAεe provided the best prognostic value over biventricular strains and added incremental value to conventional clinical predictors for patients with DCM.

**Supplementary Information:**

The online version contains supplementary material available at 10.1186/s12968-023-00967-4.

## Introduction

As dilated cardiomyopathy (DCM) eventually leads to impaired contractility, current guidelines recommend cardiac resynchronization therapy and implantable cardioverter defibrillators for improving the clinical outcome of DCM patients [1–3]. However, the risk of cardiovascular events and mortality in DCM has remained considerable [4]. Thus, accurate risk assessment and stratification are crucial in the clinical individualized management of this patient population.

Residual cardiac function represents a key determinant of long-term prognosis in DCM [1]. Traditional left ventricular ejection fraction (LVEF) is the principal measurement to assess cardiac mechanics, which only allows an estimation of global systolic function but cannot mirror differences in regional cardiac function or diastolic dysfunctions [2, 5]. Recently, cardiovascular magnetic resonance (CMR) imaging has evolved into a gold standard modality for the determination of morphology and function of heart, and feature tracking (FT) technique provides comprehensive left atrial and biventricular myocardial strain analyses for DCM patients [2, 6]. Previous studies have affirmed the feasibility and validity of CMR-FT, and multiple strain parameters showed prognostic value in DCM patients [7, 8].

Although the prognostic value of CMR-FT parameters has been established in left atrial and biventricular myocardial deformation separately, the robustness and variability of these parameters in the long-term are unknown, and the optimal strain index in DCM remains unclear. Thus, this study aimed to investigate whether multiple strain parameters assessed by CMR-FT can provide superior or combined long-term incremental prognostic information in patients with DCM.

## Methods

### Study population

**Fig. 1:**
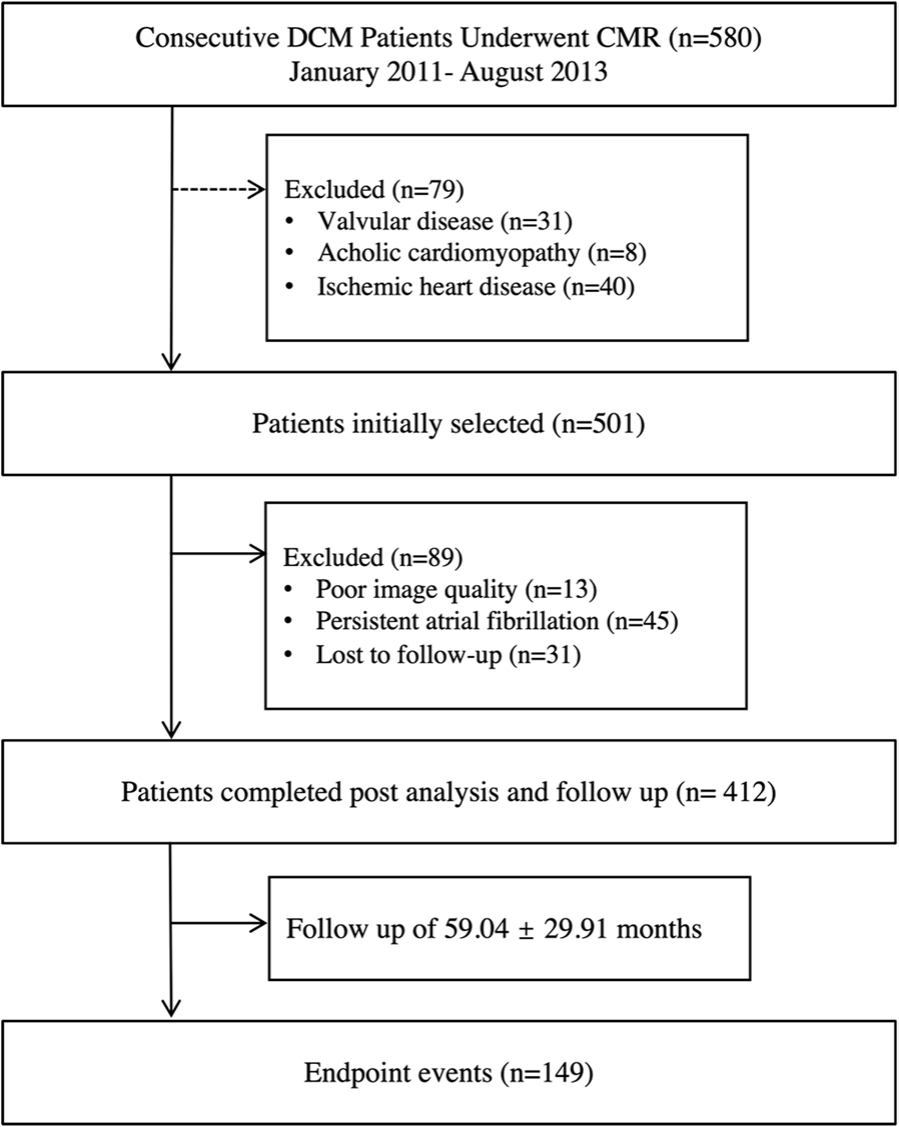
Inclusion flowchart. DCM: dilated cardiomyopathy, CMR: cardiovascular magnetic resonance

For this retrospective observational CMR-FT study, 580 consecutive DCM patients from January 2011 to August 2013 were screened for inclusion. A cohort of 412 patients underwent clinical CMR studies and was included in the final analysis (Fig. 1). Inclusion criteria were DCM diagnosed in accordance with the European Society of Cardiology/American College of Cardiology/American Heart Association committee criteria as LV end-diastolic diameters > 2 S.D. from normal and an LVEF < 50% [3, 9]. Exclusion criteria were as follows: ischemic heart disease (definite evidence of myocardial infarction or the presence of coronary artery disease, indicated by coronary artery angiography or perfusion imaging on CMR); congenital heart disease; primary valvular disease; hypertrophic cardiomyopathy; inflammatory myocardial disease; hypertensive heart disease; cardiac sarcoidosis or amyloidosis; survived cardiac arrest; scheduled for major cardiothoracic surgery; and any specific contraindication to CMR examination (pacemaker, implanted cardioverter defibrillators, cerebral aneurysm clip, orbital foreign body, claustrophobia). Patients with poor image quality for strain assessment were also excluded. Investigators performed a thorough patient interview and comprehensive review of electronic health records to document baseline medical history uniformly. Image acquisition was performed following the same protocols and post-processing was performed using standardized techniques. The study received approval from the institutional review boards of the hospital.

### CMR protocol and analysis

**Fig. 2:**
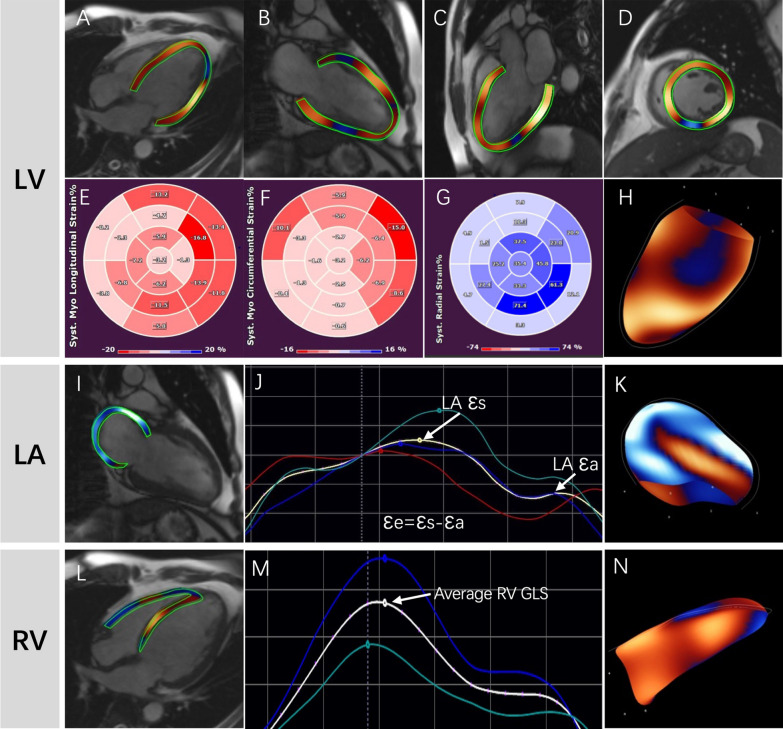
Feature tracking analysis was performed on routine cardiac cine images. **A**–**D**, **I**, **L** In long-axis and short-axis views, the endocardial and epicardial contours were automatically detected with manual correction. **E–G** The deeper red and blue indicated poor heart function of the left ventricular (LV). **J**, **M** The left atrial (LA) and right ventricular (RV) strain curves were automatically constructed. **H**, **K**, **N** Three-dimensional model of the LV, LA, and RV myocardium

All CMR scans were conducted on a 1.5 T scanner (MAGNETOM Avanto®, Siemens Healthineers). The standardized imaging protocol of our research group has previously been published in detail [10]. CMR images analyses and measurements were performed by two experienced radiologists (X.X. and Y.S. with 3 and 6 years of experience respectively) who were blinded to patient information and outcomes. LV volumes, mass, and ejection fraction were quantitatively measured from the short-axis cine images using standard techniques on local workstations. Late gadolinium enhancement (LGE) images were evaluated by using the software CVI42 (Circle Cardiovascular Imaging Inc., Calgary, Canada). LGE presence was regarded as enhancement signal in 2 phase-encoding directions and both long- and short-axis planes. The LGE pattern was classified as focal, mid-wall, subepicardial, or multiple patterns. Quantification of LGE was performed using the full width at half maximum method. FT-derived atrial and biventricular myocardial strains were detected in all patients, and endocardial and epicardial borders were semi-automatedly traced at end-diastole in short- and long-axis cine images using Qmass (Medis Medical Imaging Systems, Leiden, The Netherlands). Short-axis measurements were conducted at basal, middle, and apical levels and were derived by tracing 2-chamber, 3-chamber, as well as 4-chamber views. Papillary muscles were excluded from the LV volume. By averaging the according peak values of the segments, three-dimensional FT global longitudinal strain (GLS), global radial strain (GRS), and global circumferential strain (GCS) of LV and GLS of RV were obtained. Moreover, LA reservoir strain (εs), conduit strain (εe), and booster strain (εa) were measured at the LA end-diastolic phase in two-, three-, and four-chamber, then LA strain curves were automatedly generated (Fig. 2). Moreover, functional mitral or tricuspid regurgitation was defined as regurgitation secondary to left ventricular remodeling resulting in failure of leaflet coadaptation, in the setting of normal valve anatomy on CMR imaging [11]. Qualitative and quantitative evaluation of the mitral and tricuspid regurgitation was performed according to the EACVI position paper [12], the detailed algorithm for the grading of regurgitation severity was demonstrated in the Additional file 1: Table S1. A randomly determined cohort of 50 patients was analyzed to evaluate intra- and inter-observer variabilities.

### Follow up

Clinical endpoints were assessed via electronic health records or telephone interviews at regular intervals, using a standardized questionnaire. The primary endpoint of the study was the occurrence of major adverse cardiac events (MACE), defined as a composite of all-cause mortality, heart transplantation, and implantable cardioverter defibrillator discharge. Patient data were censored at the time of any endpoint event, and only the first event for each patient was included in the analysis. Time to event was calculated as the period between the CMR study and MACE. Mortality status was verified independently through death certificates. All event information was obtained and classified without knowledge of CMR findings.

### Statistical analysis

Descriptive characteristics were reported as frequencies and proportions for categorical variables and as mean ± SD for continuous variables. Differences between groups (patients with MACE versus without MACE) were determined by *χ*^2^ or Fisher exact tests for categorical variables and Kruskal–Wallis test for continuous variables as appropriate. The predictors of MACE were examined with univariable and multivariable Cox regression analysis. Hazard Ratios (HRs) were calculated with 95% confidence intervals. Concerning the sample size and the number of events, the confounder of age and variables with a P ≤ 0.001 in univariable analysis were included in the multivariate analysis to construct a baseline model for the prediction of MACE. Then nested Cox regression models were built to evaluate the incremental prognostic value of FT-derived strain parameters. To avoid collinearity, the most significant strain indexes of atrial and biventricular (LAεe, LVGLS, RVGLS), and combined strain indexes (LAεe + LVGLS, LAεe + LVGLS + RVGLS) were included as covariates in separate models to determine independent predictors. The variance inflation factor (VIF) test was performed in all models to avoid overfitting and multicollinearity issues (Additional file 1: Table S2). For each model, the incremental predictive power was assessed according to the chi-square value by using omnibus tests. Reclassification of patients by adding strain parameters to the baseline model was further evaluated by integrated discrimination improvement (IDI). The Harrell C-index was used to evaluate model performance and discrimination. Receiver operating characteristic (ROC) analysis was used to determine the optimal cutoff values of strain parameters for the prediction of MACE. Patient survival was illustrated by Kaplan–Meier curves and differences were evaluated by log-rank test. Intra- and interobserver variabilities of CMR-FT parameters were evaluated by intraclass correlation coefficients (ICC) as well as coefficients of variation. IBM SPSS Statistics 25.0 (Armonk, New York) and R 3.6.1 (The R Foundation, Ames, Iowa) were used for statistical analyses. For all tests, a *P* value < 0.05 was considered statistically significant.

## Results

### Baseline characteristics

Of 580 consecutive patients initially enrolled, 79 patients were excluded from the analysis because of failure to meet the criteria of DCM (including 31 patients with valvular disease, 8 patients with alcoholic cardiomyopathy, and 40 patients with ischemic heart disease) Thirty-one patients were lost to follow-up and 13 patients were excluded due to poor CMR image quality. The final cohort included 412 patients with a mean age was 45 ± 14.2 years (335 males). During the follow-up period of 59.0 ± 29.9 months, MACE occurred in 149 patients (36.1%). Sixty-one patients experienced a cardiac death, 70 underwent cardiac transplantation, and 18 patients with an appropriate ICD discharge. Baseline characteristics in patients with and without MACE are demonstrated in Table 1. Patients with MACE had significantly lower systolic and diastolic blood pressure levels, lower BMI, higher NYHA class, higher NT-proBNP, more dyspnea prevalence, less hypertension and hypercholesterolemia prevalence, larger LA and LV diameter, higher LVEDVI and LVESVI, decreased LVEF and higher prevalence and extent of LGE. In the CMR-FT analysis, all the strain parameters were significantly impaired in patients with events (all *P* < 0.05).

**Table 1:** Baseline characteristics

Variables	All patients (n = 412)	Patients without events (n = 263)	Patients with events (n = 149)	*P* value
Clinical data				
Age (y)	45.0 ± 14.2	44.8 ± 14.3	45.3 ± 14.0	0.570
Sex (Male)	335 (81.3)	215 (81.8)	120 (80.5)	0.762
BMI (kg/m^2^)	24.4 ± 4.1	24.9 ± 3.9	23.6 ± 4.3	< 0.001
BSA (m^2^)	1.8 ± 0.2	1.8 ± 0.2	1.8 ± 0.2	0.039
NYHA class III-IV	204 (49.5)	111 (42.2)	93 (62.4)	< 0.001
Family history	15 (3.6)	7 (2.7)	8 (5.4)	0.159
Dyspnea	293 (71.1)	173 (65.8)	120 (80.5)	0.001
Chest pain	32 (7.8)	24 (9.1)	8 (5.4)	0.171
Palpitation	185 (44.9)	113 (42.9)	72 (48.3)	0.294
Syncope	18 (4.4)	10 (3.8)	8 (5.4)	0.455
Hypertension	135 (32.8)	100 (38.0)	35 (23.5)	0.003
Hypercholesterolemia	113 (27.4)	82 (31.2)	31 (20.8)	0.023
Diabetes	61 (14.8)	36 (13.7)	25 (16.8)	0.396
Smoking	129 (31.3)	81 (30.8)	48 (32.2)	0.766
Excess alcohol	112 (27.2)	68 (25.9)	44 (29.5)	0.421
Heart rate (bpm)	78.4 ± 17.0	78.0 ± 17.0	79.1 ± 17.0	0.264
Systolic BP (mm Hg)	113 ± 17	118 ± 17	108 ± 15	< 0.001
Diastolic BP (mm Hg)	73 ± 12	75 ± 12	69 ± 11	< 0.001
NT-proBNP (pg/mL)	2244 ± 1951	1524 ± 1285	3202 ± 2255	< 0.001
Medications				
Beta-blockers	212 (51.5)	141 (53.6)	71 (47.7)	0.245
Anti-arrhythmic	252 (61.2)	141(53.6)	111 (74.5)	< 0.001
Anti-coagulation	209 (50.7)	132 (50.2)	77 (51.7)	0.772
ACEI/ARB	254 (61.7)	153 (58.2)	101 (67.8)	0.054
Diuretics	376 (91.3)	236 (89.7)	140 (94.0)	0.144
CMR conventional index				
LA diameter (mm)	40.3 ± 10.4	37.3 ± 9.1	45.9 ± 10.6	< 0.001
LV diameter (mm)	70.3 ± 9.4	68.0 ± 8.0	74.5 ± 10.3	< 0.001
LVEF (%)	25.9 ± 9.4	27.8 ± 9.2	22.6 ± 8.8	< 0.001
LVEDVI (ml/m^2^)	146 ± 58	128 ± 44.0	176 ± 65.6	< 0.001
LVESVI (ml/m^2^)	111 ± 53	94.7 ± 40.0	140 ± 60.4	< 0.001
LV mass index (g/m^2^)	62.9 ± 22	62.6 ± 21.6	63.5 ± 22.9	0.918
LGE presence	241 (58.5)	121 (46.0)	120 (80.5)	< 0.001
LGE extent (%)	6.2 ± 7.4	5.5 ± 8.1	7.2 ± 6.1	< 0.001
LGE pattern				0.135
Focal	17 (4.1)	10 (3.8)	7 (4.7)	
Midwall	143 (34.7)	76 (28.9)	67 (45.0)	
Subepicardial	15 (3.6)	6 (2.3)	9 (6.0)	
Multiple	66 (16.0)	29 (11.0)	37 (24.8)	
Mitral regurgitation				< 0.001
Mild	184 (44.7)	129 (49.0)	55 (36.9)	
Moderate	100 (24.3)	39 (14.8)	61 (40.9)	
Severe	21 (5.1)	7 (2.7)	14 (9.4)	
Tricuspid regurgitation				< 0.001
Mild	93 (22.6)	47 (17.9)	46 (30.9)	
Moderate	25 (6.1)	8 (3.0)	17 (11.4)	
Severe	4 (1.0)	3 (1.1)	1 (0.7)	
CMR feature tracking				
LAεs (%)	13.0 ± 7.8	15.4 ± 7.8	8.7 ± 5.7	< 0.001
LAεe (%)	6.4 ± 3.9	8.0 ± 3.8	3.7 ± 2.5	< 0.001
LAεa (%)	6.6 ± 5.2	7.3 ± 5.2	5.9 ± 4.2	< 0.001
LVGLS (%)	− 9.5 ± 4.8	− 11.0 ± 4.8	-6.9 ± 3.6	< 0.001
LVGCS (%)	− 10.9 ± 6.2	− 12.4 ± 6.2	-8.3 ± 5.1	< 0.001
LVGRS (%)	17.1 ± 6.6	17.8 ± 6.4	15.8 ± 6.6	0.003
RVGLS (%)	− 15.5 ± 7.4	− 17.6 ± 7.0	-11.8 ± 6.7	< 0.001

Values are presented as mean ± SD or n (%)

*P* value indicates comparison between patients without and with events

BMI: body mass index; NYHA: New York Heart Association; NT-pro BNP: N-terminal pro-hormone brain natriuretic peptide; ACEI:angiotensin converting enzyme inhibitor; ARB: angiotensin receptor blocker; LA: left atrial, LV: left ventricular; RV: right ventricular; LVEF: left ventricular ejection fraction; LVEDVI: left ventricular end diastolic volume index; LVESVI: left ventricular end systolic volume index; LGE: late gadolinium enhancement; LA εs: LA reservoir strain, LA εe: LA conduit strain, LA εa: LA booster strain; GLS: global longitudinal strain; GCS: global circumferential strain; GRS: global radial strain

### Univariate and multivariate Cox analysis

Clinical parameters including BMI, NYHA class III-IV, dyspnea, hypertension, hypercholesterolemia, systolic and diastolic BP, NT-proBNP, and CMR conventional indices including LA and LV diameter, LVEF, LVEDVI, LVESVI, mitral and tricuspid regurgitation, LGE presence, LGE extent, as well as all the FT derived strain parameters showed significant predictive associations with MACE in univariate analysis (Table 2).

**Table 2:** Univariable Cox analysis for prediction of MACE in DCM

Variables	HR	95% CI	*P* Value
Age	1.00	0.99–1.01	0.840
Sex	0.96	0.64–1.44	0.838
BMI	0.93	0.89–0.97	0.001
BSA	0.47	0.21–1.02	0.055
NYHA class III-IV	1.88	1.50–2.35	< 0.001
Family history	1.79	0.88–3.65	0.109
Dyspnea	2.00	1.33–3.00	0.001
Chest pain	0.63	0.31–1.29	0.210
Palpitation	1.18	0.85–1.62	0.320
Syncope	1.42	0.69–2.89	0.338
Hypertension	0.56	0.39–0.82	0.003
Hypercholesterolemia	0.64	0.43–0.95	0.026
Diabetes	1.23	0.80–1.89	0.347
Smoking	1.10	0.78–1.54	0.605
Excess alcohol	1.18	0.83–1.68	0.351
Heart rate	1.00	0.99–1.01	0.450
Systolic BP	0.97	0.96–0.98	< 0.001
Diastolic BP	0.96	0.94–0.97	< 0.001
NT-proBNP	1.47	1.37–1.58	< 0.001
Beta-blockers	0.78	0.57–1.08	0.138
Anti-arrhythmic	2.12	1.47–3.06	< 0.001
Anti-coagulation	1.04	0.76–1.44	0.790
ACEI/ARB	1.36	0.96–1.91	0.083
Diuretics	1.64	0.83–3.21	0.152
LA diameter	1.07	1.05–1.08	< 0.001
LV diameter	1.03	1.01–1.05	0.001
LVEF	0.95	0.93–0.97	< 0.001
LVEDVI	1.01	1.01–1.02	< 0.001
LVESVI	1.01	1.01–1.02	< 0.001
LV mass index	1.00	1.00–1.01	0.346
LGE presence	3.74	2.49–5.62	< 0.001
LGE extent	1.02	1.00–1.04	0.027
LGE pattern	1.15	0.96–1.38	0.120
Mitral regurgitation	2.14	1.78–2.57	< 0.001
Tricuspid regurgitation	1.59	1.31–1.94	< 0.001
LAεs	0.89	0.86–0.92	< 0.001
LAεe	0.69	0.65–0.74	< 0.001
LAεa	0.93	0.90–0.96	< 0.001
LVGLS	1.21	1.16–1.27	< 0.001
LVGCS	1.12	1.08–1.16	< 0.001
LVGRS	0.96	0.94–0.99	0.003
RVGLS	1.11	1.08–1.14	< 0.001

HR: Hazard Ratio; CI: confidence intervals; other abbreviations as in Table 1

In the multivariate Cox analysis, a baseline model of conventional variables including age, NYHA class III-IV, NT-proBNP, LVEF, and LGE presence was constructed. Then after adjustment for the baseline variables, LAεe, LVGLS, and RVGLS were all independently associated with MACE in each strain-based model. For every 1% increase in LAεe, the risk of MACE decreased by 0.25 times, and for every 1% decrease in LVGLS and RVGLS, the risk of MACE increased by 0.12 and 0.02 times respectively. Importantly, in the combined strain models, LAεe still showed independent prognostic value over LVGLS, RVGLS, and conventional clinical and imaging factors (Table 3).

**Table 3:** Multivariable Cox analysis for prediction of MACE in DCM

Variables	LAεe model		LVGLS model		RVGLS model		LAΕE + LVGLS model		LAεe + LVGLS + RVGLS model	
	HR(95% CI)	*P* Value	HR(95% CI)	*P* Value	HR(95% CI)	*P* Value	HR(95% CI)	*P* Value	HR(95% CI)	*P* value
Age	0.99 (0.97–1.00)	0.015	0.99 (0.98–1.00)	0.061	1.00 (0.98–1.01)	0.353	0.99 (0.97–1.00)	0.015	0.99 (0.97–1.00)	0.016
NYHA class III-IV	1.03 (0.72–1.46)	0.869	1.00 (0.69–1.43)	0.984	1.08 (0.76–1.55)	0.668	1.04 (0.73–1.48)	0.839	1.04 (0.73–1.48)	0.839
NT-proBNP	1.25 (1.15–1.36)	< 0.001	1.35 (1.25–1.47)	< 0.001	1.32 (1.22–1.44)	< 0.001	1.25 (1.15–1.36)	< 0.001	1.25 (1.15–1.36)	< 0.001
LVEF	0.98 (0.96–1.01)	0.118	0.99 (0.96–1.02)	0.397	0.98 (0.96–1.00)	0.101	0.98 (0.96–1.01)	0.119	0.98 (0.96–1.01)	0.121
LGE presence	2.80 (1.84–4.27)	< 0.001	3.37 (2.21–5.13)	< 0.001	3.10 (2.04–4.73)	< 0.001	2.79 (1.83–4.25)	< 0.001	2.79 (1.83–4.27)	< 0.001
LAεe	0.75 (0.70–0.80)	< 0.001	n/a	n/a	n/a	n/a	0.74 (0.68–0.80)	< 0.001	0.74 (0.68–0.81)	< 0.001
LVGLS	n/a	n/a	1.12 (1.06–1.17)	< 0.001	n/a	n/a	0.99 (0.93–1.04)	0.723	0.99 (0.94–1.05)	0.743
RVGLS	n/a	n/a	n/a	n/a	1.06 (1.03–1.09)	< 0.001	n/a	n/a	0.10 (0.97–1.03)	0.979

HR: Hazard Ratio; CI: confidence intervals; n/a: Not applicable, other abbreviations as in Table 1

### Incremental prognostic value

**Fig. 3:**
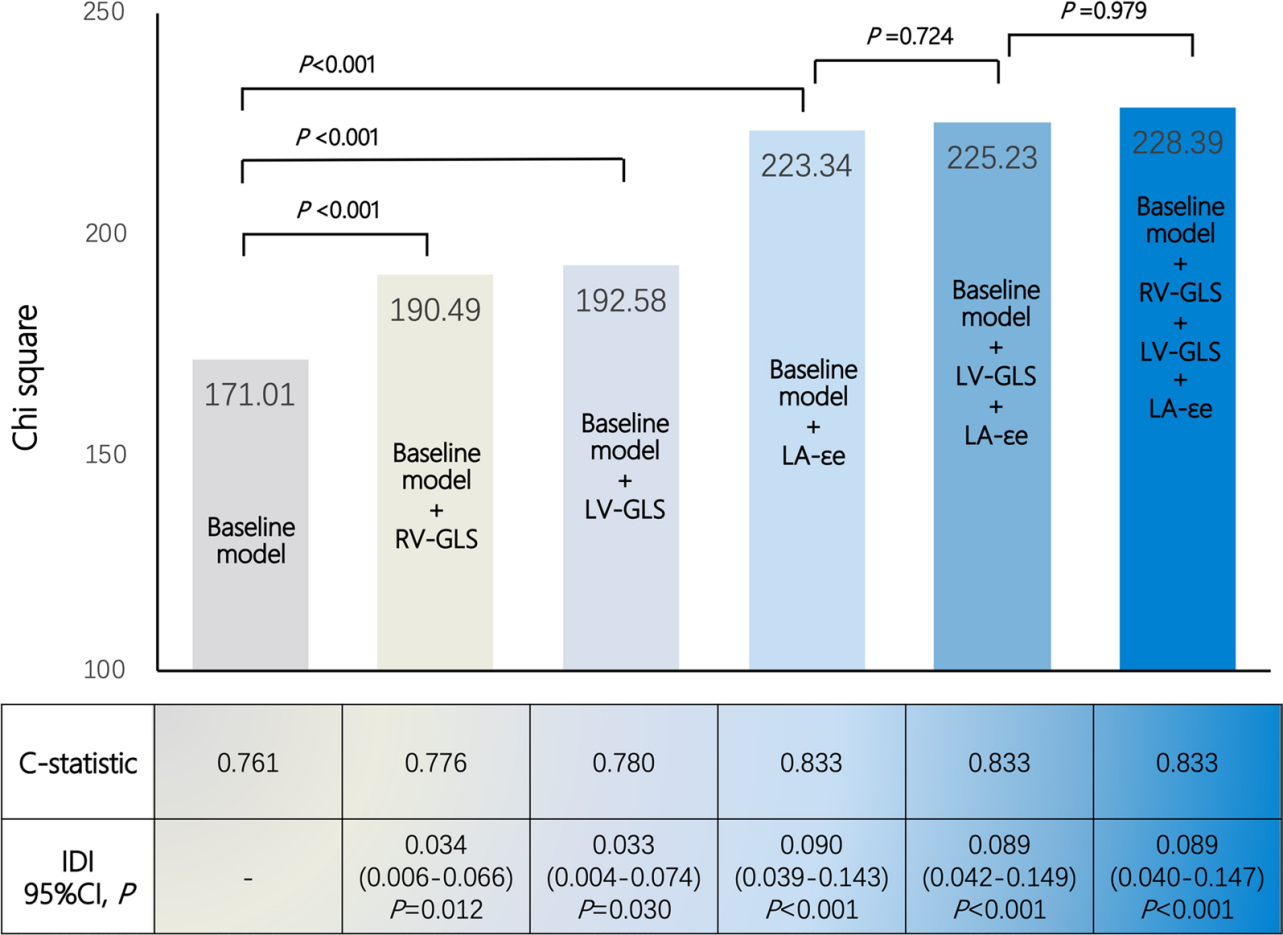
Incremental prognostic value of strain parameters for dilated cardiomyopathy (DCM). Variables in the baseline model include age, NYHA class III–IV, NT-proBNP, LVEF, and LGE presence. Integrated discrimination improvement (IDI) is used to judge improvement in model performance between the strain-based models and the baseline model

The chi-square values of RVGLS, LVGLS, and LAεe based models were 190.49, 193.58, and 223.34 respectively. Stepwise inclusion of RV, LV, or LA strain had incremental prognostic values in predicting MACE compared with conventional clinical predictors (all, *P* < 0.001, Fig. 3). However, the Chi-square values of the combined LA + LV and LA + LV + RV strain models were 225.23 and 228.39 respectively, indicating no incremental prognostic value beyond LAεe based model (*P* = 0.724, 0.979, respectively).

In reclassification analysis, the addition of LAεe to baseline variables resulted in a significant increase in the C-statistic (from 0.76 to 0.83, *P* < 0.001) and an IDI of 0.09 (95%CI 0.042–0.149, *P* < 0.001). The addition of LVGLS and RVGLS also showed a significant reclassification of patients versus the baseline model (C-statistic 0.78, *P* < 0.001; IDI 0.034, 95%CI 0.006–0.066, *P* = 0.012; C-statistic 0.78, *P* < 0.001; IDI 0.033, 95%CI 0.004–0.074, *P* = 0.03; respectively). Notably, the addition of combined LAεe + LVGLS or LAεe + LVGLS + RVGLS also led to a reclassification of patients versus the baseline model but had no significant increase to the LAεe model (Fig. 3).

### Survival analysis

**Fig. 4:**
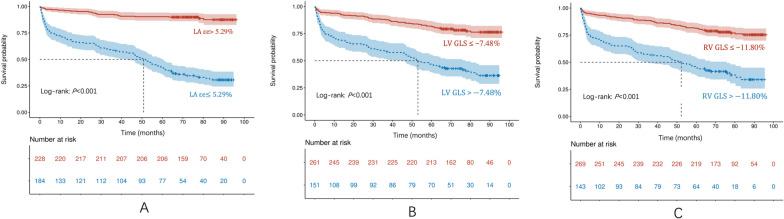
Kaplan–Meier survival curves of dilated cardiomyopathy (DCM) stratified by the optimal cutoff values of strain parameters. Patients with LAεe ≤ 5.29%, LVGLS > − 7.48%, or RVGLS > − 11.80% were associated with a significantly higher rate of events (all log-rank *P* < 0.001)

LAεe with an optimal cutoff value of 5.29% showed the best prediction of MACE (AUC = 0.854). Cutoff values of LVGLS and RVGLS for the prediction of MACE were − 7.48% and − 11.80% (AUC = 0.754, 0.733, respectively). The correlation between strain parameters and conventional CMR indices was illustrated in detail with a heatmap (Additional file 1: Fig. S1). Consequently, in the Kaplan–Meier analysis, patients with a LAεe ≤ 5.29%, LVGLS > − 7.48%, or RVGLS > − 11.80% had significantly higher rate of adverse events (all log-rank* P* < 0.001, Fig. 4).

**Fig. 5:**
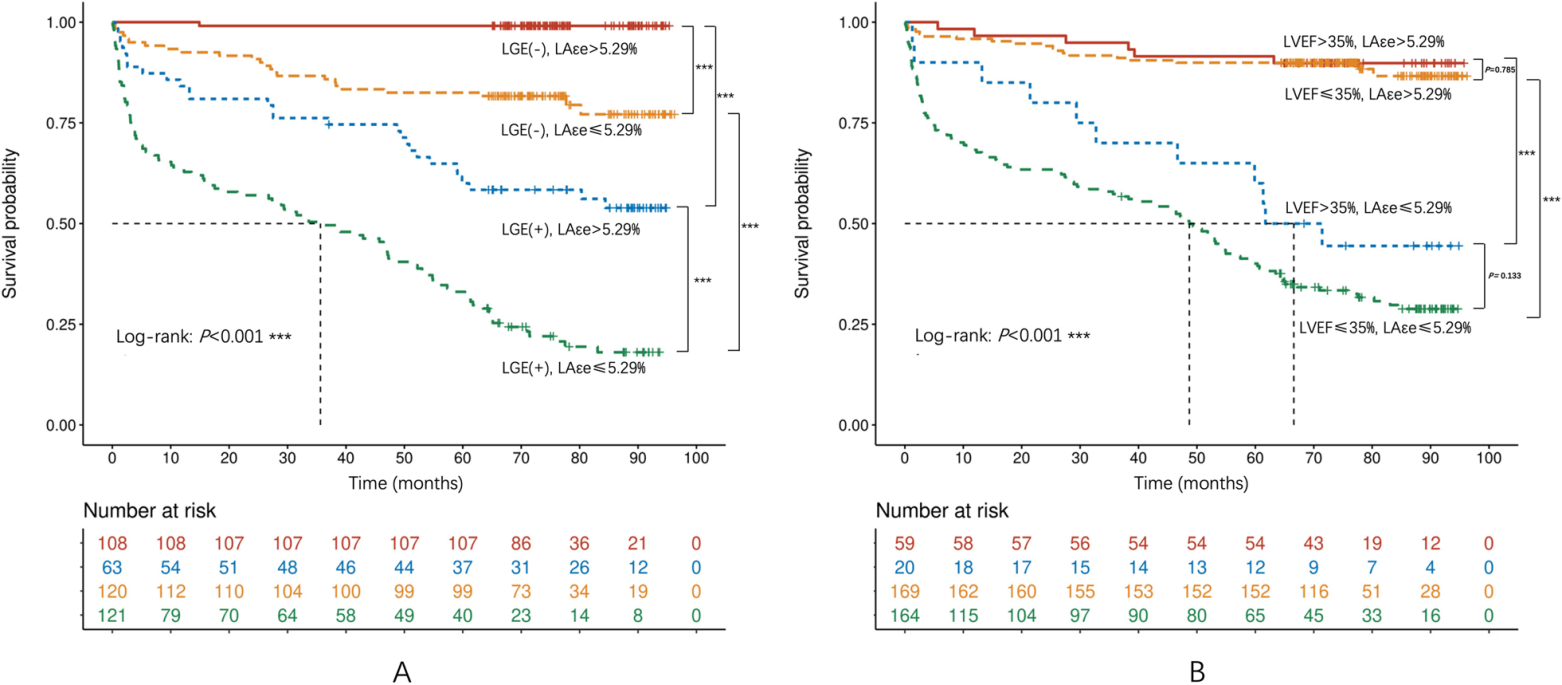
Kaplan–Meier survival curves for subgroups of dilated cardiomyopathy (DCM). The rate of events was significantly higher in those with impaired LAεe, irrespective of LGE presence or LVEF (all log-rank *P* < 0.001). However, LVEF did not affect survival probability in patients with preserved or impaired LAεe (log-rank *P* = 0.785, 0.133)

In the additional subgroup analyses, the Kaplan–Meier curves showed that patients with impaired LAεe had lower survival probability, irrespective of positive or negative LGE presence. Likewise, in patients with severely reduced LVEF (< 35%), the rate of adverse events was also significantly higher in those with impaired LAεe (all log-rank *P* < 0.001, Fig. 5, Additional file 1: Fig. S2).

### Reproducibility analysis

Quantification of CMR-FT derived atrial and biventricular strain parameters had good reproducibility in DCM patients, as all the intra- and interobserver intraclass correlation coefficients were higher than 0.75 and with low standard error of measurement (Additional file 1: Table S3).

## Discussion

This study provides unique real-world long-term evidence for the use of CMR-FT to predict the outcomes of DCM. To the best of our knowledge, this is the first study to assess LA, LV, and RV myocardial strain dysfunction at the same time, revealing that the LAεe had superior prognostic value than biventricular strains and was incremental to conventional clinical predictors. The inclusion of myocardial strain components in the predictive algorithm may help guide clinical management decisions and further monitor risk events for patients with DCM.

### Pathophysiological basis of myocardial strain

Relaxation and contractile impairment of LV is the major pathologic change of DCM, and reversal of LV reverse remodeling is regarded as a key therapeutic goal [1]. In addition to LV wall thinning and dilation, increased LV filling pressures, and reduced ventricular compliance, adverse remodeling characteristics in DCM also include the enlargement of other chambers [2, 13, 14].

Long-axis function plays a fundamental role in cardiac mechanics, contributing to ventricular ejection by reducing LV cavity size as the mitral annulus is pulled toward the apex [15, 16]. Biventricular dysfunction has been reported in DCM [7, 14], however, impaired LA function and morphologic alteration have emerged as more powerful predictors of adverse cardiovascular events in patients with DCM [13, 17, 18]. Myocardial pathological processes of DCM not merely directly affect the LA myocardial wall and decrease compliance, but also reduce the diastolic atrial-ventricular pressure gradient and further worsen the heart failure symptoms [6, 13]. Of note, the LA conduit strain reflects an emptying of blood from the LA during early ventricular diastole, reductions in LA conduit strain are dominantly thought to represent accompanying changes in ventricular relaxation and myocardial stiffness, resulting in increased pressure and ensuing dilatation of the LA, causing a decrease of atrial compliance and impaired contractile function in the late ventricular diastole [6, 13]. Therefore, LA conduit strain, which reflects the passive filling of LV is likely to be the first and most sensitive strain index affected by LV dysfunction. Our study, accordingly, validated that LAεe was more closely associated with MACE in DCM patients.

### CMR-FT for assessment of myocardial strain

CMR has evolved into a major tool for diagnostic and prognostic assessment of patients with DCM by providing data on morphology, function, perfusion, viability, and tissue characterization [2, 19]. It offers greater accuracy and reproducibility for the measurement of myocardial strain, allowing serial assessment of the progression of disease or treatment response in individual patients [19]. Although echocardiographic methods have also been applied to assess myocardial strain, there are still limitations due to foreshortening, lower reproducibility of acquisition planes, limited temporal resolution when arrhythmia, and difficulties assessing circumferential and radial strains, which can be overcome by CMR derived strains [5, 15, 20].

CMR-FT technology shows promise in allowing measurement of myocardial strain in the clinical setting. Importantly, this approach can be applied to routine cine CMR images, thus avoiding the need for dedicated additional sequences and is easy to implement in practice [20, 21]. All strain parameters in our study thereby showed high repeatability. Compared with other techniques for the detection of myocardial strain [20, 22], CMR is capable of better visualizing both ventricular and atrial structures, and CMR-FT can easily and sensitively derive quantifiable markers of LA function and remodeling. Moreover, LA strains are reliable and accurate indexes that, besides systolic function, reflect LV diastolic function [13]. Specifically, LA εs is modulated by LV volume, εe is influenced by LV relaxation and early diastolic properties, and εa is dependent on LV end-diastolic pressures and compliance [23].

### Prognostic value of CMR-FT

Compared with previous studies [7, 8, 16], the incidence of adverse events in this study was higher, which is probably because of the significantly reduced LVEF (mean 25.9 ± 9.4%) indicating that most patients in this study had advanced DCM. Despite guideline-directed medication being used for this population, as a tertiary center of cardiovascular diseases, most patients transferred to our hospital suffered from severe symptoms, and the therapeutic effects were relatively poor. Regardless of this selection bias, our study demonstrated that CMR-FT provided important independent and incremental prognostic information in DCM patients, as LAεe, LVGLS, and RVGLS all significantly improved reclassification versus conventional clinical data and imaging features in each strain-based model. The addition of LAεe to the baseline model provided the best discrimination, confirming the nonnegligible important role of diastolic dysfunction in DCM patients.

Notably, when combining atrial and biventricular strains with conventional variables, the LVGLS and RVGLS were not prognostic in the multivariable Cox model. This is probably because LA function and especially LA conduit strain, can be more sensitive than ventricular size and volume change, and better reflect overall function [6, 13, 23]. As such, it is reasonable to consider LA strain as a more sensitive indicator. Recent studies have also stated the superior or incremental prognostic value of LA strain over a few common clinical and imaging markers [24, 25], and our study further elucidated the role of LAεe beyond left and right ventricular strain.

It is interesting to note that LVEF was not significantly associated with MACE after adjusting for strain parameters in multivariable models. Moreover, in subgroup survival analysis, irrespective of LVEF, the rate of events was significantly higher in those with decreased LAεe, that is, the outcomes of DCM patients can be further worsened by impaired LA strain. Although current guidelines recommending implantable cardioverter device (ICD) placement based primarily on an LVEF < 35% [26], we found LV function to be a poor discriminator of clinical outcome risk [27]. Recent studies have shown that patients with reduced LVEF are a heterogeneous group with variability in the mortality risk [28–30], and DCM is a dynamic disease with left ventricular remodeling that cannot be evaluated by LVEF alone. Buxton et al. [28] found that patients with decreased or preserved LVEF had a similar percentage of arrhythmic deaths, and Gorgels et al. [29] demonstrated the majority of patients with mortality had an LVEF > 30% in the Maastricht registry of circulatory death. Consequently, there is a need to migrate from conventions embedded in present clinical practice that continue to place central emphasis on a crude and solitary marker of LV function. Our study demonstrates the potential clinical importance of using LA strain for further risk stratification in DCM. Collectively, the incorporation of LAεe into overall clinical risk scores may better risk-stratify and deliver personalized care for patients with DCM.

## Limitations

Our study has several limitations. First, as a single-center retrospective study, external validation to confirm the generalizability of CMR-FT is desirable and the relevant thresholds of myocardial strain need verification in a larger cohort. Second, as a CMR study, there is a degree of selection bias related to being able to undergo a CMR or enhancement imaging examination, resulting in the exclusion of patients with severe symptoms, large body size, contrast allergy, severe renal impairment, or severe claustrophobia. Second, 7.5% loss to follow-up in this cohort was a potential limitation of survival estimates, which may also lead to bias. Third, the high number of clinical and imaging parameters evaluated in our study can potentially result in false discovery. Moreover, the CMR-FT strain assessment is dependent on reader experience which is subject to observer variability. Futhermore, various software packages may result in discrepancies; thus, the applicability of our findings to other FT vendors requires further investigation. Fourth, we did not perform a genetic characterization of study patients and no conclusions can be drawn about a possible correlation between DCM associated genes and phenotype. Also, this study lacked T1 mapping imaging data and quantitative RVEF which needs to be supplemented in further investigation. Future prospective studies are required to validate our results and to address the value of LA strain in DCM phenotyping, prognostication, and management.

## Conclusion

LAεe provided the best prognostic value over biventricular strains and added incremental value to conventional clinical predictors for patients with DCM.

### Supplementary Information

**Additional file 1: Figure S1. A** Receiver operating characteristic analysis of strain parameters for differentiation major adverse clinical event (MACE) in dilated cardiomyopathy (DCM). **B** Correlogram illustrates cardiovascular magnetic resonance (CMR) conventional index and strain parameters. Blue indicates a positive correlation and red indicates a negative correlation. The darker the color, the higher the correlation between the two variables. **Figure S2. A** Kaplan–Meier survival curves stratified by LVEF, LGE, and LAεe. **B** Events rate in different groups classified by LVEF, LGE, and LAεe. The LAεe was transformed into categories according to the optimal cutoff value calculated by receiver operating characteristic analysis. **Figure S3.** Bootstrapped distribution of estimated receiver operating characteristic (ROC) curve for predicting MACE in DCM patients. The mean optimal cutoff values for (A) LA εe, (B) LV GLS and (C) RV GLS were about 5.3%, -7.5%, and -11.8% respectively. **Table S1.** Evaluation of MR and TR. **Table S2.** Variance inflation factor measurements. **Table S3.** Inter- and intra- observer variability of strain parameters. **Table S4.** Comparison of CMR measurements between patients included and lost to follow-up.

## Data Availability

The datasets used and/or analyzed during the current study are available from the corresponding author upon reasonable request.

## Abbreviations

CMR: Cardiovascular magnetic resonance
DCM: Dilated cardiomyopathy
FT: Feature tracking
GCS: Global circumferential strain
GLS: Global longitudinal strain
GRS: Global radial strain
ICD: Implantable cardioverter device
IDI: Integrated discrimination improvement
LA: Left atrial
LAεa: LA booster strain
LAεe: LA conduit strain
LAεs: LA reservoir strain
LGE: Late gadolinium enhancement
LV: Left ventricular
LVEF: Left ventricular ejection fraction
MACE: Major adverse cardiac event
RV: Right ventricular
