# Supplementary material for: Incremental prognostic value of left atrial and biventricular feature tracking in dilated cardiomyopathy: a long-term study
Source: J Cardiovasc Magn Reson. 2023 Dec 7;25:76. doi: 10.1186/s12968-023-00967-4 (PMC10701983; doi:10.1186/s12968-023-00967-4)
Supplement: Supplementary file 1 — Additional file 1: Figure S1. A Receiver operating characteristic analysis of strain parameters for differentiation major adverse clinical event (MACE) in dilated cardiomyopathy (DCM). B Correlogram illustrates cardiovascular magnetic resonance (CMR) conventional index and strain parameters. Blue indicates a positive correlation and red indicates a negative correlation. The darker the color, the higher the correlation between the two variables. Figure S2. A Kaplan–Meier survival curves stratified by LVEF, LGE, and LAεe. B Events rate in different groups classified by LVEF, LGE, and LAεe. The LAεe was transformed into categories according to the optimal cutoff value calculated by receiver operating characteristic analysis. Figure S3. Bootstrapped distribution of estimated receiver operating characteristic (ROC) curve for predicting MACE in DCM patients. The mean optimal cutoff values for (A) LA εe, (B) LV GLS and (C) RV GLS were about 5.3%, -7.5%, and -11.8% respectively. Table S1. Evaluation of MR and TR. Table S2. Variance inflation factor measurements. Table S3. Inter- and intra- observer variability of strain parameters. Table S4. Comparison of CMR measurements between patients included and lost to follow-up. [file 12968_2023_967_MOESM1_ESM.docx]

**Additional files**

**
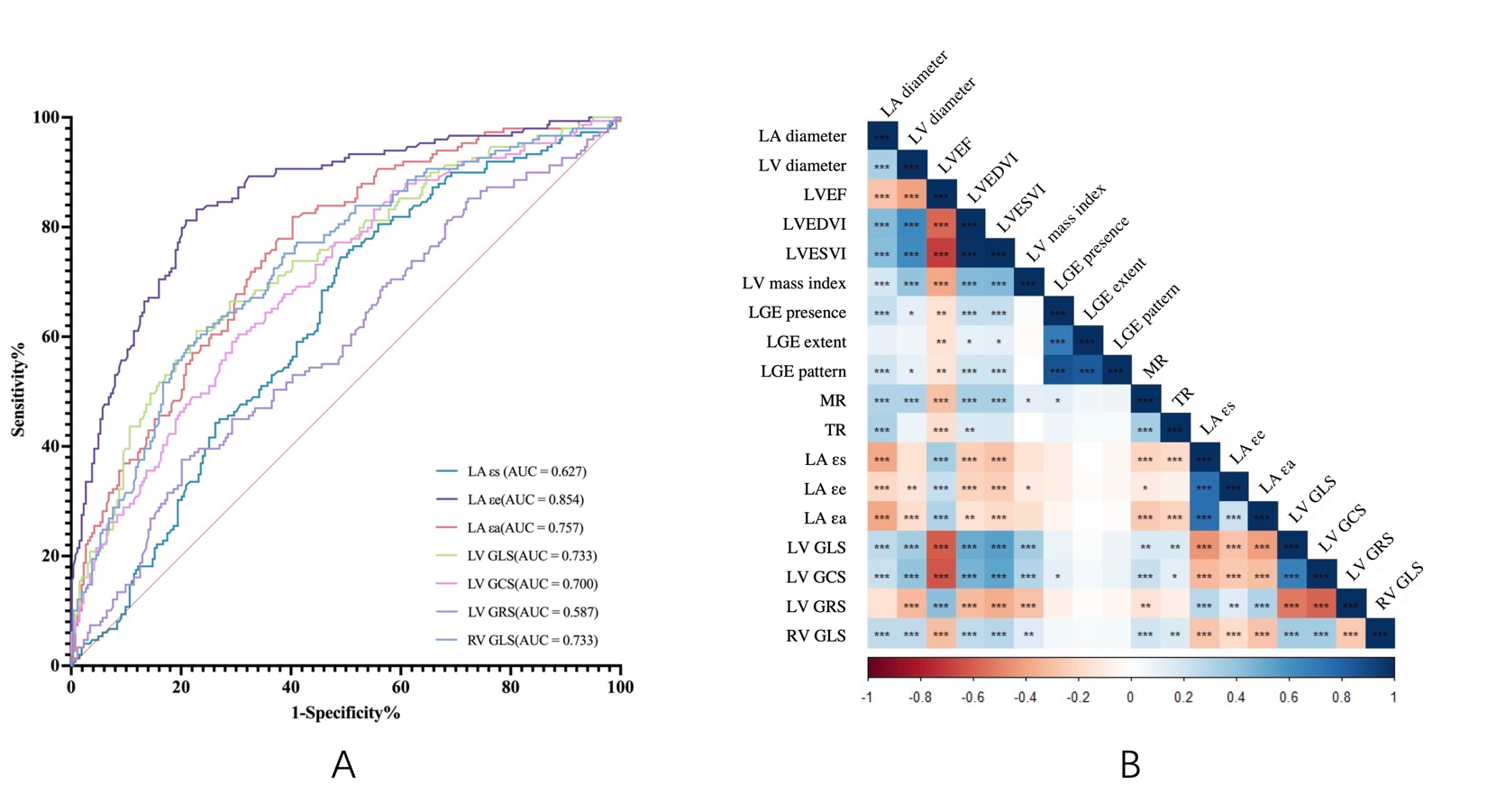
**

**Figure S1** **A** Receiver operating characteristic analysis of strain parameters for differentiation major adverse clinical event (MACE) in dilated cardiomyopathy (DCM). **B** Correlogram illustrates cardiovascular magnetic resonance (CMR) conventional index and strain parameters. Blue indicates a positive correlation and red indicates a negative correlation. The darker the color, the higher the correlation between the two variables.


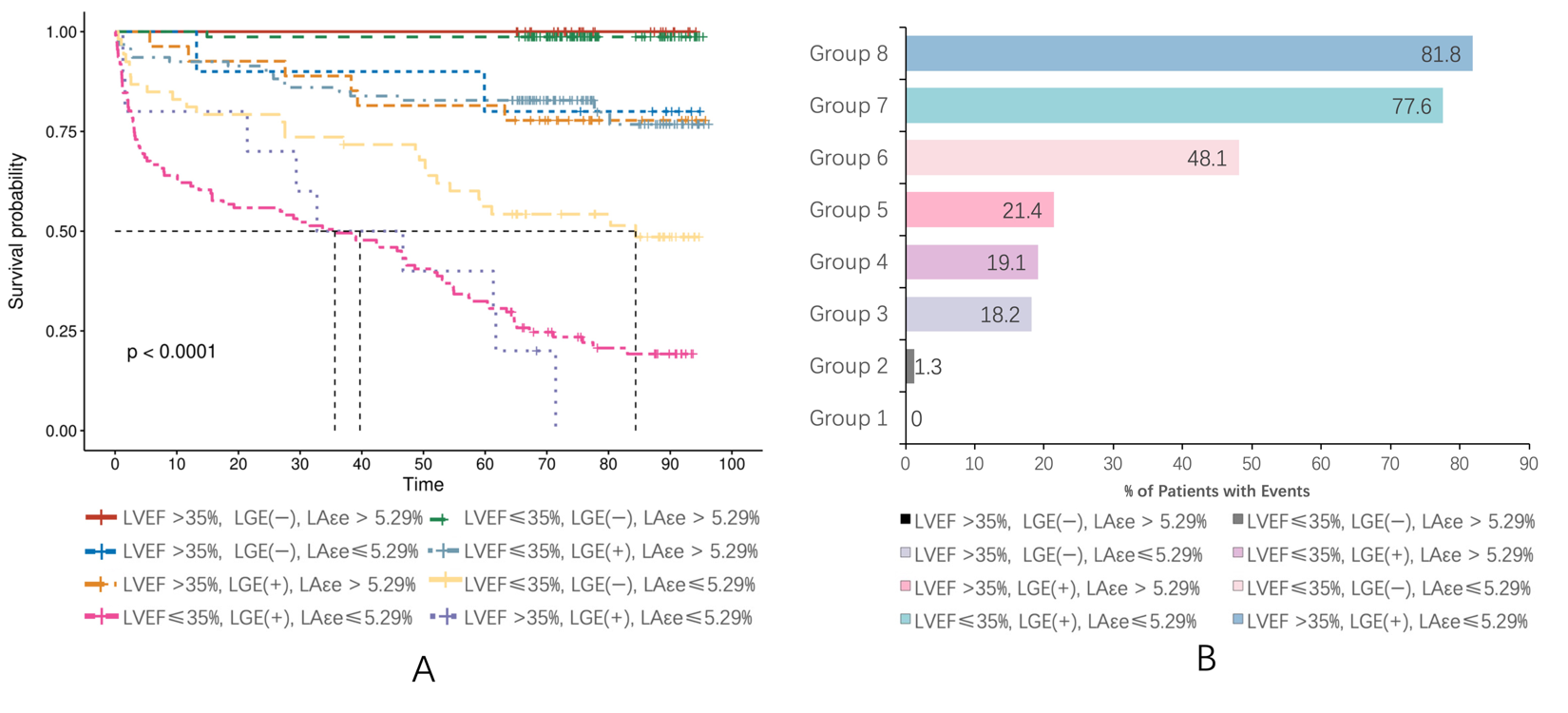


**Figure S2** **A** Kaplan-Meier survival curves stratified by LVEF, LGE, and LAεe. **B** Events rate in different groups classified by LVEF, LGE, and LAεe. The LAεe was transformed into categories according to the optimal cutoff value calculated by receiver operating characteristic analysis.


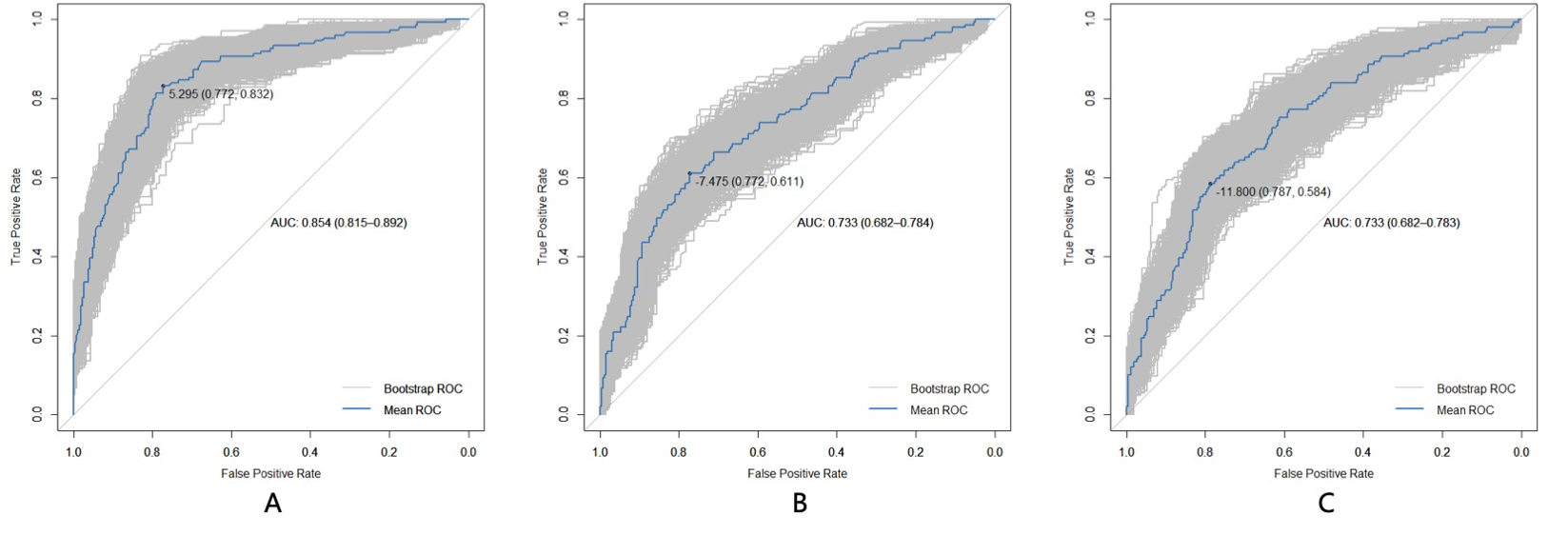


**Figure S3** Bootstrapped distribution of estimated receiver operating characteristic (ROC) curve for predicting MACE in DCM patients. The mean optimal cutoff values for (A) LA εe, (B) LV GLS and (C) RV GLS were about 5.3%, -7.5%, and -11.8% respectively.

| **Table S1** Evaluation of MR and TR | | | | |
| --- | --- | --- | --- | --- |
| **Grading of severity** | | **EROA (mm^2^)** | **R Vol (mL)** | **RF (%)** |
| MR | Mild | < 20 | < 30 | < 30 |
|  | Moderate | 20-39 | 30-59 | 30-49 |
|  | Severe | ≥40 | ≥60 | ≥50 |
|  |  |  |  |  |
| TR | Mild | < 20 | < 30 | ≤15 |
|  | Moderate | 20-39 | 30-44 | 30-49 |
|  | Severe | ≥40 | ≥45 | ≥50 |
| MR: mitral regurgitation; TR: tricuspid regurgitation; EROA: effective regurgitant orifice area; R Vol: regurgitant volume; RF: regurgitant fraction. | | | | |

| **Table S2** Variance inflation factor measurements | | | | | | |
| --- | --- | --- | --- | --- | --- | --- |
| **VIF of Variables** | **Baseline  Model** | **RVGLS  Model** | **LVGLS  Model** | **LAεe Model** | **LVGLS + LAεe Model** | **LVGLS + RVGLS + LAεe Model** |
| Age | 1.04 | 1.07 | 1.07 | 1.09 | 1.09 | 1.12 |
| NYHA class III-IV | 1.16 | 1.16 | 1.19 | 1.12 | 1.13 | 1.13 |
| NT-proBNP | 1.09 | 1.16 | 1.13 | 1.19 | 1.19 | 1.22 |
| LVEF | 1.16 | 1.29 | 1.38 | 1.15 | 1.43 | 1.45 |
| LGE presence | 1.05 | 1.07 | 1.07 | 1.06 | 1.06 | 1.08 |
| RVGLS | n/a | 1.29 | n/a | n/a | n/a | 1.85 |
| LVGLS | n/a | n/a | 1.37 | n/a | 1.85 | 2.06 |
| LAεe | n/a | n/a | n/a | 1.17 | 1.54 | 1.70 |
| A VIF above 4 indicates that multicollinearity might exist, a VIF higher than 10 indicates significant multicollinearity.  VIF: variance inflation factor; other abbreviations as in Table 1. | | | | | | |

| **Table S3** Inter- and intra- observer variability of strain parameters | | | | | |
| --- | --- | --- | --- | --- | --- |
| Strain Parameters | Intra-Observer | |  | Inter-Observer | |
|  | ICC | 95% CI |  | ICC | 95% CI |
| LAεs | 0.887 | 0.793 - 0.901 |  | 0.857 | 0.743 - 0.897 |
| LAεe | 0.913 | 0.842 - 0.934 |  | 0.893 | 0.811 - 0.923 |
| LAεa | 0.892 | 0.865 - 0.973 |  | 0.864 | 0.803 - 0.962 |
| LVGLS | 0.916 | 0.812 - 0.968 |  | 0.902 | 0.786 - 0.961 |
| LVGCS | 0.905 | 0.834 - 0.977 |  | 0.886 | 0.754 - 0.958 |
| LVGRS | 0.897 | 0.815 - 0.988 |  | 0.867 | 0.723 - 0.974 |
| RVGLS | 0.915 | 0.778 - 0.956 |  | 0.898 | 0.732 - 0.923 |
| ICC: intraclass correlation coefficients; CI: confidence intervals; other abbreviations as in Table 1. | | | | | |

| **Table S4** Comparison of CMR measurements between patients included and lost to follow-up | | | |
| --- | --- | --- | --- |
| **Variables** | **Patients Included**  **(n=412)** | **Patients Lost to Follow-up**  **(n=31)** | ***P* value** |
| CMR conventional index |  |  |  |
| LA diameter (mm) | 40.3 ± 10.4 | 38.7 ± 6.3 | **0.450** |
| LV diameter (mm) | 70.3 ± 9.4 | 66.9 ± 8.1 | **0.114** |
| LVEF (%) | 25.9 ± 9.4 | 26.7 ± 7.8 | **0.550** |
| LVEDVI (ml/m^2^) | 146 ± 57.6 | 143 ± 28.7 | **0.455** |
| LVESVI (ml/m^2^) | 111 ± 53.0 | 108 ± 30.3 | **0.677** |
| LV mass index (g/m^2^) | 62.9 ± 22.0 | 60.0 ± 12.6 | **0.755** |
| LGE presence | 241 (58.5) | 15 (48.4) | **0.272** |
| LGE extent (%) | 6.2 ± 7.5 | 7.7 ± 9.2 | **0.799** |
| Mitral regurgitation | 299 (72.6) | 18 (58.1) | **0.113** |
| Tricuspid regurgitation | 121 (29.4) | 7 (22.6) | **0.487** |
| CMR feature tracking |  |  |  |
| LAεs (%) | 13.0 ± 7.8 | 14.3 ± 4.0 | **0.060** |
| LAεe (%) | 6.4 ± 3.9 | 7.2 ± 3.7 | **0.189** |
| LAεa (%) | 6.6 ± 5.2 | 7.0 ± 3.3 | **0.120** |
| LVGLS (%) | -9.5 ± 4.8 | -9.3 ± 2.9 | **0.913** |
| LVGCS (%) | -10.9 ± 6.2 | -10.5 ± 4.4 | **0.898** |
| LVGRS (%) | 17.1 ± 6.6 | 17.0 ± 4.4 | **0.941** |
| RVGLS (%) | -15.5 ± 7.4 | -16.0 ± 5.4 | **0.479** |
| Values are presented as mean ± SD or n (%).  LA: left atrial; LV: left ventricular; RV: right ventricular; LVEF: left ventricular ejection fraction; LVEDVI: left ventricular end-diastolic volume index; LVESVI: left ventricular end-systolic volume index; LGE: late gadolinium enhancement; LA εs: LA reservoir strain, LA εe: LA conduit strain, LA εa: LA booster strain; GLS: global longitudinal strain; GCS: global circumferential strain; GRS: global radial strain. | | | |
